# From Susceptibility to Severity: The Impact of Interleukin-33 rs1929992 Polymorphism on Asthma in a Taiwanese Population

**DOI:** 10.3390/life16091395

**Published:** 2026-08-24

**Authors:** Te-Chun Hsia, Liang-Wen Hang, Te-Chun Shen, Jie-Long He, Kai-Ling Huang, Ding-Han Chen, Yun-Chi Wang, Da-Tian Bau

**Affiliations:** 1 Division of Pulmonary and Critical Care Medicine, Department of Internal Medicine, China Medical University Hospital, Taichung 404327, Taiwan; 2 Terry Fox Cancer Research Laboratory, Department of Medical Research, China Medical University Hospital, Taichung 404327, Taiwan; 3 School of Medicine, College of Medicine, China Medical University, Taichung 404333, Taiwan; 4 Division of Critical Care Medicine, Chu Shang Show Chwan Hospital, Nantou 55782, Taiwan; 5 Department of Post-Baccalaureate Veterinary Medicine, Asia University, Taichung 413305, Taiwan; 6 Graduate Institute of Biomedical Sciences, China Medical University, Taichung 404333, Taiwan; 7 Office of Research and Development, Asia University, Taichung 413305, Taiwan

**Keywords:** age, asthma, gender, genotype, interleukin-33, polymorphism, severity, Taiwan

## Abstract

Asthma is a chronic inflammatory airway disease strongly influenced by genetic factors. Interleukin-33 (*IL-33*), a key mediator of type 2 immune responses, has been implicated in airway inflammation and remodeling. However, the effects of *IL-33* polymorphisms on asthma susceptibility and severity remain unclear, particularly in East Asian populations. This study investigated five *IL-33* polymorphisms (rs1891385, rs16924159, rs12551256, rs1929992, and rs7044343) in relation to asthma risk and severity in Taiwanese individuals. A total of 198 asthmatic patients and 453 age- and sex-matched controls were enrolled. Genotypes were determined using PCR-RFLP. Odds ratios (ORs) and 95% confidence intervals (CIs) were calculated to assess associations with asthma susceptibility and severity. Among the five polymorphisms, only rs1929992 was significantly associated with asthma. Compared with the TT genotype, CT and CC carriers had reduced asthma risk (OR = 0.61 and 0.51, respectively). The dominant model (CT+CC) also showed a protective effect (OR = 0.58, 95%CI = 0.40–0.82, *p* = 0.0032), which was supported by allelic analysis (OR = 0.70, 95%CI = 0.55–0.89, *p* = 0.0044). Among the five polymorphisms, rs1929992 was associated with asthma susceptibility. Under the dominant model, CT + CC carriers showed lower odds of asthma than TT carriers (OR = 0.58, 95% CI = 0.40–0.82, *p* = 0.0032), and the association remained significant after correction for multiple testing. Exploratory stratified analyses identified associations in the younger and male subgroups, while exploratory severity analysis suggested lower odds of more severe asthma among variant genotype carriers. These secondary findings should be considered hypothesis-generating because of the reduced subgroup sample sizes. Overall, rs1929992 was associated with asthma susceptibility in this Taiwanese population; however, independent replication and functional studies are required before its biological or clinical relevance can be established.

## 1. Introduction

Asthma is a heterogeneous chronic respiratory disorder characterized by persistent airway inflammation, reversible airflow obstruction, bronchial hyperresponsiveness, and progressive airway remodeling [1,2]. Recent epidemiological evidence indicates that approximately 300 million individuals worldwide are affected by asthma, and this burden is expected to rise substantially, with an additional 100 million cases projected in the coming years [3,4]. Given its increasing prevalence and considerable healthcare impact, elucidating the determinants of asthma susceptibility remains a major research priority. In addition to several well-established triggers, including airborne allergens [5,6], ambient fine particulate matter (PM2.5) [7,8], and tobacco smoke exposure [9,10], accumulating evidence has highlighted the critical contribution of host genetic background to asthma development. Numerous polymorphisms located within immune-regulatory genes have been implicated in disease predisposition and clinical outcomes. Among these, genetic variations in *IL4* and its corresponding receptor [11,12,13], as well as *IL-13* [14,15], have consistently been associated with altered immune responses and asthma risk. However, the association of other inflammatory-related genes with asthma remained largely unrevealed.

Asthmatic inflammation is orchestrated by a complex network of immune and airway-resident structural cells, contributing substantially to the heterogeneous clinical manifestations of the disease. Type 2-driven inflammation represents the predominant endotype, being present in most pediatric patients and in more than half of adults with asthma [16]. This inflammatory pattern is typically characterized by allergic sensitization, eosinophilic infiltration in both peripheral blood and airways, excessive mucus production, and elevated circulating IgE concentrations [16]. Among the mediators implicated in type 2 immunity, interleukin-33 (*IL-33*) has emerged as a pivotal upstream regulator of asthmatic inflammation since accumulated clinical evidence has demonstrated that elevated *IL-33* expression is closely associated with disease severity in pediatric and/or adult populations [17,18,19,20]. Elevated levels of *IL-33* have been detected in asthmatic airways and are positively correlated with worsening clinical manifestations. Mechanistically, enhanced *IL-33* expression within bronchial epithelial cells and airway smooth muscle cells has been linked to augmented airway hyperresponsiveness, a hallmark feature of asthma [21]. Furthermore, patients with severe asthma exhibit an increased abundance of *IL-33*-responsive group 2 innate lymphoid cells (ILC2s) [18]. Additional support for the pathogenic role of *IL-33* derives from transcriptomic studies. Alternative splicing of the *IL-33* gene can generate a biologically active isoform that retains signaling capability and is released extracellularly [22]. Genetic investigations have further reinforced the contribution of *IL-33* to asthma susceptibility. Multiple genome-wide association studies have consistently identified *IL-33* polymorphisms as significant genetic determinants of asthma risk across diverse populations [23,24,25]. In contrast, naturally occurring loss-of-function variants within the *IL-33* locus have been associated with reduced circulating eosinophil counts and a lower prevalence of asthma [26]. Experimental studies using animal models have provided complementary mechanistic evidence. Disruption of *IL-33* signaling leads to a reduction in basal eosinophil levels and markedly attenuates allergen-induced eosinophilic airway inflammation and airway hyperresponsiveness [21,27,28,29]. In summary, evidence from clinical, genetic, and experimental studies converge to indicate *IL-33* as a potential central regulator of asthma pathogenesis.

**Figure 1:**
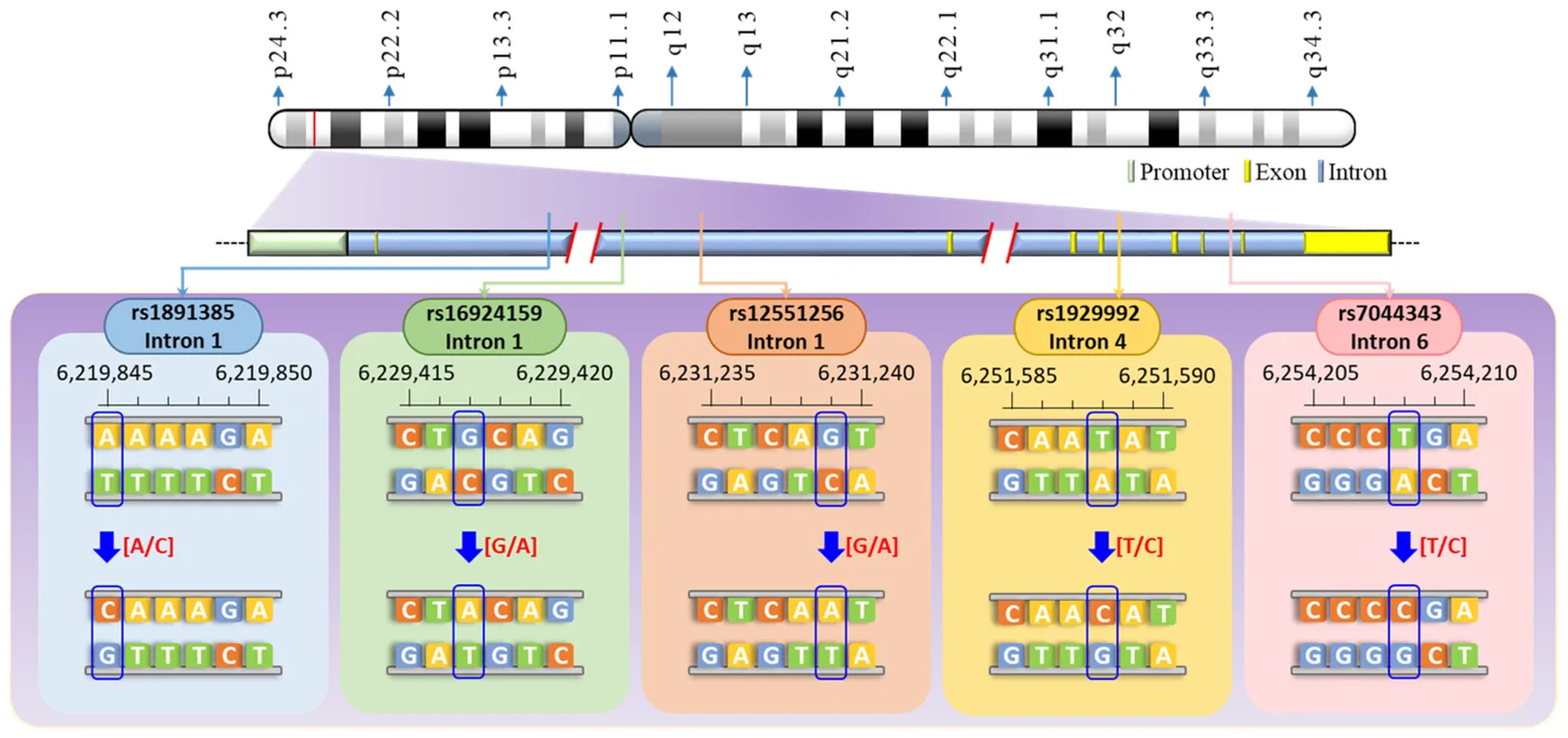
The physical map information for the nearby sequences of *IL-33* rs1891385, rs16924159, rs12551256, rs1929992, and rs7044343 polymorphic sites. The rs1891385, rs16924159, rs12551256, rs1929992, and rs7044343 are located in introns 1, 1, 1, 4, and 6, respectively.

Several polymorphisms within the *IL-33* gene, including rs1891385, rs16924159, rs12551256, rs1929992, and rs7044343, have been investigated in relation to susceptibility to various human diseases. These five polymorphisms were selected for the present candidate-gene study based on their previously reported disease associations and their distribution across different intronic regions of the *IL-33* gene. The present study focused on selected potentially relevant *IL-33* variants rather than comprehensive coverage or fine mapping of the entire *IL-33* locus. Although additional *IL-33* polymorphisms have been associated with asthma susceptibility or *IL-33* expression, these variants were beyond the scope of the original genotyping panel. However, despite the well-established role of *IL-33* in type 2 inflammation, immune regulation, and airway remodeling, the contribution of these genetic variants to asthma remains incompletely understood, particularly in East Asian populations. Therefore, the present study aimed to investigate the associations of *IL-33* rs1891385, rs16924159, rs12551256, rs1929992, and rs7044343 polymorphisms with asthma susceptibility in a Taiwanese case–control population (Figure 1). Additionally, we explored whether these variants are associated with asthma severity, thereby providing further insight into the potential genetic contribution of *IL-33* to asthma susceptibility and clinical severity.

## 2. Materials and Methods

### 2.1. Recruitment of Asthmatic Patients and Control Participants

This case–control study enrolled 198 patients with physician-diagnosed asthma from China Medical University Hospital and 453 age- (±5 years) and sex-matched non-asthmatic controls, consistent with our previous study design [30,31]. Asthma diagnoses were independently confirmed by at least two board-certified pulmonologists under the supervision of Dr. Hsia. Disease classification and severity assessment followed the Global Initiative for Asthma (GINA) recommendations [32]. Patients were subsequently stratified into four severity categories based on clinical symptoms, attack frequency, and medication requirements. Clinical and demographic data for all participants are summarized in Table 1.

### 2.2. DNA Extraction and Sample Preservation

Genomic DNA was extracted from peripheral blood buffy coat fractions obtained from all participants, with sample processing performed within 24 h of collection to ensure optimal DNA integrity [33,34]. Following isolation, DNA samples were preserved at −80 °C for long-term storage. Genomic DNA from both asthma cases and non-asthmatic controls was quantified, normalized to uniform concentrations, aliquoted into working stocks, and systematically organized according to previously established laboratory protocols [30,35].

### 2.3. Genotyping of IL-33 Polymorphisms

The genotypic profiling of the *IL-33* polymorphisms was performed using polymerase chain reaction (PCR) followed by restriction fragment length polymorphism (RFLP) analysis. Briefly, target DNA fragments encompassing the polymorphic site of rs1891385, rs16924159, rs12551256, rs1929992, and rs7044343 were amplified using PCR. The resulting PCR products were then digested with the restriction endonucleases *HpyCH4* V (rs1891385 and rs16924159), *Dde* I, *Ssp* I and *BsoB* I, respectively (New England Biolabs, Taipei, Taiwan). The information about the locations, paired primer sequences, the corresponding restriction endonucleases, and the differential fragments based on the genotypes for each polymorphic site was summarized in Table 2. For rs1929992, the T/C allele designation used in the present PCR-RFLP assay corresponds to the complementary A/G notation reported in several previous studies (T=A and C=G on the opposite strand). PCR-RFLP was selected because the present investigation was a targeted candidate-gene association study involving five predefined *IL-33* polymorphisms rather than a genome-wide or sequencing-based analysis. This approach provides a practical and cost-effective method for genotyping a limited number of known variants in a moderate-sized study population. For quality control, the overall genotyping call rate was 100%. All the genotyping process was conducted independently by at least two researchers in a double-blind manner to ensure accuracy. Notably, all genotyping results achieved a 100% success rate, with complete concordance across all samples. There was no sample yielding ambiguous restriction patterns.

### 2.4. Statistical Analysis Approach

Hardy–Weinberg equilibrium (HWE) for *IL-33* polymorphisms in the control group was evaluated using the Chi-square goodness-of-fit test to verify genetic stability and population representativeness. Differences in demographic variables, including age and sex, as well as genotype and allele distributions between asthma cases and controls were analyzed using Pearson’s Chi-square test with appropriate 2 × 2 or 2 × 3 contingency tables. Associations between *IL-33* polymorphisms and asthma susceptibility were assessed using logistic regression to calculate crude odds ratios (ORs) and corresponding 95% confidence intervals (CIs). Multivariable logistic regression analyses were additionally performed with adjustment for age, sex, and smoking status to calculate adjusted odds ratios (aORs) and corresponding 95% CIs. In addition, genotype-phenotype relationships under an additive genetic model were assessed using the Cochran-Armitage trend test, while a dominant model was also examined. Post hoc power analyses were performed for the association of *IL-33* rs1929992 with asthma susceptibility in the overall population and in the age- and sex-stratified subgroups. Power was estimated based on the observed sample sizes and genotype distributions under the dominant model (CT+CC vs. TT). All statistical tests were two-sided. To account for multiple comparisons across the five investigated *IL-33* polymorphisms, Bonferroni correction was applied, resulting in a corrected significance threshold of *p* < 0.01 (0.05/5). Both nominal and Bonferroni-adjusted *p*-Values are reported for the primary analyses. Age-, sex-, and asthma severity-stratified analyses were considered exploratory and were interpreted cautiously because of the additional comparisons and reduced subgroup sample sizes.

## 3. Results

### 3.1. Comparison of Demographic and Clinical Profiles Between Asthmatic and Control Groups

Table 1 presents the demographic and clinical characteristics of 198 asthma patients and 453 non-asthmatic controls, including age, gender distribution, pulmonary function parameters, and disease severity. Owing to the intentional frequency matching design, no statistically significant differences were observed in age or gender between the two groups (*p* = 0.2972 and 0.9956, respectively). In contrast, lung function indices were markedly impaired in the asthma cohort, as reflected by significantly lower FEV_1_/FVC ratios and reduced percent-predicted FEV_1_ values (both *p* < 0.0001). With respect to clinical severity stratification, asthmatic patients were categorized into four stages, comprising 30.3% in stage 1, 32.8% in stage 2, 17.2% in stage 3, and 19.7% in stage 4 (Table 1).

### 3.2. Contribution of IL-33 Genotypes to Asthma Risk

The genotypic distributions of *IL-33* rs1891385, rs16924159, rs12551256, rs1929992, and rs7044343 polymorphisms among 198 asthma patients and 453 non-asthmatic controls are summarized in Table 3. In the control population, all five polymorphisms conformed to HWE for rs1891385, rs16924159, rs12551256, rs1929992, and rs7044343 polymorphisms (*p* = 0.1976, 0.2925, 0.8448, 0.4619, and 0.1300, respectively), supporting genetic stability of the study cohort. No significant differences in genotype or allele distributions were observed for rs1891385, rs16924159, rs12551256, or rs7044343 under any of the evaluated genetic models (all *p* > 0.05). After Bonferroni correction for the five investigated polymorphisms (corrected significance threshold, *p* < 0.01), the association of rs1929992 with asthma susceptibility remained significant under the dominant model (CT+CC vs. TT, *p* = 0.0032) and in the Cochran-Armitage trend test (*p* = 0.0036). In contrast, the individual CT and CC genotype comparisons did not reach the corrected significance threshold.

### 3.3. Association Between IL-33 Allelic Frequencies and Asthma Risk

To further examine the role of the *IL-33* rs1891385, rs16924159, rs12551256, rs1929992, and rs7044343 polymorphisms in asthma susceptibility, we analyzed the allelic frequencies, as presented in Table 4. A significant difference was observed in the frequency of the variant C allele at *IL-33* rs1929992 between asthmatic and non-asthmatic individuals (OR = 0.70, 95%CI = 0.55–0.89, *p* = 0.0044). The association of the rs1929992 C allele with lower odds of asthma remained statistically significant after Bonferroni correction (nominal *p* = 0.0044; Bonferroni-adjusted *p* = 0.0220). There was no such significance found as for any variant allele at other polymorphic sites comparing with their corresponding wild-type allele (all *p* > 0.05, Table 4).

### 3.4. Stratified Analysis of IL-33 rs1929992 Genotypes in Relation to Age and Gender

To further explore the interaction between *IL-33* rs1929992 genotypes and age or gender in determining asthma susceptibility, asthmatic patients were categorized into younger (25–40) and elder (>40) age groups as shown in Table 5. The results of stratified analysis showed that there were significant differential distributions of the variant CT and CC genotypes between asthmatic and non-asthmatic individuals in the 25–40 age subgroup (OR = 0.51 and 0.39, 95%CI = 0.33–0.82 and 0.21–0.72, *p* = 0.0073 and 0.0040). However, no difference in the distributions of *IL-33* rs1929992 genotypes were observed among individuals elder than 40 years (*p* for trend = 0.7071 and all *p* > 0.05, Table 5). Regarding gender, there were significant differential distributions of the variant CT and CC genotypes between asthmatic and non-asthmatic individuals among males (*p* for trend = 0.0019), but not among females (*p* for trend = 0.2400 and all *p* > 0.05, Table 6). In detail, there were significant differential distributions of the variant CT and CC genotypes between asthmatic and non-asthmatic individuals among males (OR = 0.44 and 0.34, 95%CI = 0.25–0.79 and 0.16–0.77, *p* = 0.0082 and 0.0107, Table 6). Post hoc power analysis showed an estimated statistical power of 84.8% for the overall association of rs1929992 under the dominant model. The corresponding power estimates were 90.5% for the 25–40 age subgroup and 89.3% for the male subgroup. Nevertheless, because stratification substantially reduced the effective sample sizes, these secondary analyses should be regarded as exploratory and hypothesis-generating. The observed differences between age or sex strata should not be interpreted as evidence of true biological interaction and require confirmation in larger independent cohorts.

### 3.5. Stratified Analysis of IL-33 rs1929992 Genotypes in Relation to Asthma Symptom Severity

We further investigated the relationship between *IL-33* rs1929992 genotypes and asthma severity by categorizing asthmatic patients into two groups, milder (stages 1 and 2) and severer asthma (stages 3 and 4) groups. The genotype distributions of *IL-33* rs1929992 among these two subgroups are summarized in Table 7. A significant difference in genotype distribution was observed between patients with milder and severer asthma (*p* for trend = 0.0024, Table 7). Specifically, the frequency of the CC genotype was significantly lower in the severer asthma group (7.7%) than in the milder asthma group (21.0%), corresponding to a 77% reduction in the likelihood of severe asthma when compared with the TT genotype (OR = 0.23, 95%CI = 0.08–0.65, *p* = 0.0075). Although the CT genotype was also less frequent among patients with severer asthma (41.5%) than among those with milder asthma (47.4%), the association did not reach statistically significant level (OR = 0.55, 95%CI = 0.29–1.04, *p* = 0.0893). When the CT and CC genotypes were combined, carriers of the variant genotypes exhibited a significantly lower risk of severe asthma compared with individuals carrying the TT genotype, with frequencies of 49.2% and 68.4%, respectively (OR = 0.45, 95%CI = 0.24–0.82, *p* = 0.0140). Furthermore, under the recessive genetic model, subjects carrying the CC genotype showed a significantly reduced susceptibility to severe asthma compared with those harboring the TT+CT genotypes (7.7% versus 21.0%, OR = 0.31, 95%CI = 0.11–0.85, *p* = 0.0303, Table 7). In the exploratory severity analysis, carriers of the rs1929992 variant genotypes had lower odds of severe asthma. However, this finding should be interpreted cautiously because of the relatively small subgroup size and requires independent validation.

## 4. Discussion

Several clinical studies conducted across different ethnic populations have consistently demonstrated that *IL-33* expression is significantly elevated in asthmatic patients compared with healthy controls, supporting its pivotal role in asthma pathogenesis. In a North African cohort, Hamzaoui and his colleagues reported that both induced sputum and serum *IL-33* concentrations were markedly increased in Tunisian children with asthma compared with healthy controls, and that *IL-33* levels further increased with disease severity, suggesting a close relationship between *IL-33*-mediated inflammation and clinical activity [36]. In 2018, Charrad and his colleagues observed significantly higher serum *IL-33* levels in another cohort of Tunisian pediatric asthmatics and further demonstrated an association between elevated *IL-33* expression and the rs1342326 polymorphism, indicating that genetic variation may influence *IL-33* production and asthma susceptibility [37]. This was the first study to report that the overexpression of *IL-33* was associated with *IL-33* various genotypes. In Middle Eastern populations, Momen and his colleagues showed that Iranian asthmatic patients exhibited substantially higher circulating *IL-33* levels than controls, while serum *IL-33* was inversely correlated with FEV1, supporting a role for *IL-33* in airway obstruction and disease severity [38]. Likewise, Rabea and his colleagues reported significantly elevated serum *IL-33* concentrations among Egyptian asthmatic patients and identified a strong positive correlation between *IL-33* levels and asthma severity [39]. Comparable findings have also been reported in European populations. Gluck and his colleagues demonstrated increased *IL-33* levels in both serum and exhaled breath condensate from Polish asthma patients [40]. In a relatively large Lithuanian cohort, Gasiuniene and his colleagues found significantly elevated serum *IL-33* concentrations in asthmatic patients, with the highest levels observed in allergic and eosinophilic phenotypes [41]. In 2024, Yang and his colleagues confirmed in Chinese children that serum *IL-33* concentrations were significantly higher in asthmatic subjects than in healthy controls and were negatively associated with pulmonary function parameters, indicating that *IL-33* may serve as a useful diagnostic and disease-monitoring biomarker in pediatric asthma [42]. Taken together, despite differences in ethnicity, age distribution, specimen type, and analytical methodology, all the seven studies consistently demonstrated significantly elevated *IL-33* levels in asthma patients relative to healthy controls.

Several studies have investigated the associations of *IL-33* polymorphisms with asthma susceptibility and disease severity across different ethnic populations, although the reported findings remain heterogeneous and inconclusive. Previous studies have commonly reported rs1929992 using the complementary A/G allele notation, corresponding to the T/C designation used in the present study. The earliest evidence was provided by Chen and his colleagues in a Chinese population, demonstrating that the rs928413 G allele was significantly associated with an increased risk of atopic asthma, suggesting that this promoter-region variant may influence *IL-33* expression and predispose individuals to allergic airway inflammation [43]. Subsequently, Schröder and his colleagues reported that both rs928413 and rs1342326 were associated with an increased risk of hay fever in a large European birth cohort and further showed that carriers of the variant alleles exhibited reduced frequencies of regulatory T cells and elevated suppressor of cytokine signaling 3 (SOCS3) expression, indicating a potential mechanism through dysregulated immune tolerance [44]. Among the *IL-33* polymorphic variants investigated, rs1342326 has emerged as one of the most extensively studied loci. Charrad et al. demonstrated in Tunisian children that the rs1342326 C allele and AC/CC genotypes were associated with a significantly reduced risk of asthma development. Interestingly, despite its protective effect against asthma susceptibility, the C allele was more frequently observed among severe asthmatic patients and was associated with elevated serum *IL-33* concentrations, suggesting a complex role in disease progression [37]. Similarly, Matloubi et al. reported that the rs1342326 CC genotype increased asthma risk in an Iranian population and was associated with atopic, mild, and eosinophilic asthma phenotypes, whereas rs1342326 was linked to moderate-to-severe asthma, indicating that different *IL-33* polymorphisms may influence distinct clinical manifestations of the disease [45]. In contrast, Ahmadi and his colleagues failed to detect a significant association between rs1342326 and asthma susceptibility in another Iranian cohort, despite observing significantly elevated serum *IL-33* levels among asthmatic patients, emphasizing the inconsistency of genetic findings across populations [46]. In the present study, *IL-33* rs1342326 was also genotyped in the study cohort. However, all examined Taiwanese participants were found to carry the AA genotype, and no genetic variation was detected at this locus. This observation is largely consistent with large-scale population data available from the NCBI database, in which the major A allele exhibits an extremely high frequency of 99.97% among East Asian populations [47]. The near-complete fixation of the A allele in East Asians may explain the absence of polymorphic variation in our cohort. Rabea and his colleagues found no significant association between rs1929992 and asthma susceptibility in Egyptian patients [39]. Similar negative genetic findings were reported in Turkish and Iraqi populations, where rs1929992 showed no significant contribution to asthma risk despite markedly elevated circulating *IL-33* concentrations in asthmatic subjects [48,49]. In a Brazilian population, Queiroz and his colleagues identified rs12551256 as a protective variant, with the G allele significantly reducing asthma susceptibility [50]. Genotyping analysis of this polymorphic locus was also performed in the present study. However, no significant association was observed between the genotype distributions and asthma susceptibility or disease severity (Table 3 and Table 4). Interestingly, a Finnish prospective birth cohort study by Teräsjärvi and his colleagues suggested that rs12551268 may confer protection against childhood asthma, although the association did not reach statistical significance [51]. To sum up, although elevated *IL-33* expression has been consistently observed in patients with asthma and is widely recognized as a key contributor to disease pathogenesis, investigations focusing on *IL-33* genetic polymorphisms remain relatively limited. Moreover, the available evidence is fragmented across multiple genetic loci, resulting in an incomplete understanding of the contribution of *IL-33* genetic variants to asthma susceptibility and clinical manifestations. Additional large-scale multicenter studies incorporating diverse populations are warranted to clarify the contribution of *IL-33* genetic variants to asthma susceptibility and clinical severity.

The first notable finding of the present study was the identification of a significant difference in the genotypic and allelic distributions of *IL-33* rs1929992 between asthmatic patients and non-asthmatic controls in the Taiwanese population. In contrast, no such association was observed for the other examined *IL-33* polymorphisms, including rs1891385, rs16924159, rs12551256, and rs7044343 (Table 3 and Table 4). Interestingly, the association observed in the present Taiwanese population differs from previous studies conducted in Egyptian, Turkish, and Iraqi populations, in which no significant relationship between rs1929992 and asthma susceptibility was identified [39,48,49]. This discrepancy is unlikely to be explained by ethnicity alone. Population-specific differences in allele frequencies and local linkage disequilibrium architecture may influence the extent to which rs1929992 captures a functional signal within the *IL-33* locus. In our Taiwanese controls, the rs1929992 C-allele frequency was 48.12%, closely approximating the East Asian reference frequency of 49.96%, whereas lower frequencies have been reported in several other ancestral populations (Table 8). Environmental factors may also contribute to heterogeneous genetic associations, as exposure to tobacco smoke, ambient air pollution, allergens, and occupational agents differs geographically and may modify asthma susceptibility. Furthermore, differences in participant age, asthma phenotype, disease severity, control selection, sample size, and analytical approaches across studies may influence the observed effect estimates. Thus, the present findings should not be interpreted as evidence of a uniquely Taiwanese biological effect of rs1929992. Instead, they suggest a potentially population-dependent association that requires replication in larger multiethnic cohorts incorporating genetic ancestry, local linkage disequilibrium structure, and relevant environmental exposures.

The biological mechanism underlying the association between rs1929992 and asthma susceptibility remains uncertain. Because rs1929992 is located within an intronic region of *IL-33*, it does not directly alter the amino acid sequence of the encoded protein. Further interrogation of publicly available functional genomic resources, including GTEx and ENCODE, did not identify established evidence supporting rs1929992 as a significant *IL-33* eQTL or a functionally validated regulatory variant. Available annotations likewise do not currently establish a direct effect of rs1929992 on *IL-33* transcription. Nevertheless, intronic variants may influence gene regulation through effects on chromatin accessibility, enhancer activity, transcription factor binding, RNA processing, or other regulatory mechanisms. Alternatively, rs1929992 may serve as a marker for another functional variant within the *IL-33* locus through linkage disequilibrium. Therefore, the present association should not be interpreted as evidence that rs1929992 itself directly alters *IL-33* expression or function. Fine-mapping of the *IL-33* locus, tissue-specific regulatory analyses, and experimental functional studies will be required to determine whether rs1929992 has direct regulatory activity or instead tags another causal variant.

We also explored the association of rs1929992 with asthma susceptibility according to age and sex. Specifically, the CT and CC genotypes of rs1929992 were significantly associated with reduced asthma susceptibility in the younger (25 to 40 years) subgroup, whereas no significant effect was detected in the elder (older than 40 years) subgroup (Table 5). It should be emphasized that the current cohort was restricted to adult subjects aged ≥ 25 years (Table 1), which may influence the observed stratified effects. Previous epidemiological and genetic studies have predominantly focused on pediatric asthma, particularly in genome-wide association analyses, while adult-onset asthma has received comparatively less attention [53,54,55]. However, accumulating evidence indicates that pediatric and adult asthma differ substantially in terms of etiological factors, clinical manifestations, therapeutic responses, and disease control strategies [56,57,58]. Consistent with this concept, asthma heterogeneity across age groups has been further supported by subtype classification studies identifying five pediatric and four adult asthma endotypes [59]. Accordingly, refinement of disease subtyping and treatment response profiling is essential for the advancement of precision medicine approaches in asthma management [60]. Although an association was observed in the younger subgroup but not in the older subgroup (Table 5), this difference should not be interpreted as evidence of an age-specific genetic effect. Given the reduced sample sizes after stratification, the present age-stratified findings are exploratory and hypothesis-generating and require confirmation in adequately powered independent cohorts. Regarding sex-specific effects, although gender-related differences in asthma prevalence and pathophysiology have been increasingly recognized [61,62,63], genotype-based investigations remain limited and often yield inconsistent results. In the present study, a significant association between rs1929992 and asthma was observed in males but not in females (Table 6). However, this difference should not be interpreted as evidence of a sex-specific effect because of the limited subgroup sample sizes. Accordingly, this observation should be regarded as hypothesis-generating rather than evidence of a true sex-dependent genetic effect. Formal interaction analyses in substantially larger cohorts will be required to evaluate possible effect modification by sex.

An additional exploratory observation was the association between *IL-33* rs1929992 and asthma severity, with variant genotypes being less frequent among patients classified as having more severe disease (Table 7). Severe asthma accounts for approximately 4–10% of the asthmatic population and is clinically characterized by increased morbidity, poorer prognosis, and a greater need for individualized therapeutic strategies [64,65]. Within this exploratory analysis, carriers of the rs1929992 variant, particularly those with the CC genotype, showed lower odds of more severe asthma. However, because the severity analysis involved a relatively small number of patients and multiple secondary comparisons, these findings should be regarded as hypothesis-generating and require independent confirmation. Validation in larger, multi-ethnic cohorts is necessary to confirm the robustness and generalizability of this association. Moreover, the relationship between *IL-33* rs1929992 and disease severity may be influenced by the criteria used for severity classification; therefore, future studies should aim to standardize severity definitions and assessment frameworks. Such efforts will be essential for clarifying genotype-phenotype correlations and clarifying the potential relationship between *IL-33* genetic variation and asthma severity.

Several limitations of the present study should be acknowledged. First, this was a single-center, hospital-based case–control study conducted in a Taiwanese population, and the moderate sample size, particularly after stratification by age, sex, and asthma severity, may limit the precision and generalizability of the findings. Furthermore, although all participants were recruited from a Taiwanese population, subtle population stratification cannot be completely excluded because ancestry-informative markers or genome-wide genetic data were not available. Second, although age, sex, and smoking status were considered in the multivariable analyses, residual confounding cannot be excluded. Information regarding other potentially relevant clinical and environmental factors, including atopy and occupational exposure, was not systematically available for all participants and therefore could not be incorporated into the adjusted models. Third, although correction for multiple testing was applied to the primary analyses, the age-, sex-, and severity-stratified analyses involved substantially smaller sample sizes and were exploratory. These secondary findings should therefore be regarded as hypothesis-generating rather than evidence of established subgroup-specific biological effects. Fourth, an independent replication cohort, particularly one including other ethnic populations, was not available. Therefore, the reproducibility and generalizability of the association between rs1929992 and asthma require further confirmation. Fifth, functional experiments were not performed to determine whether rs1929992 directly affects *IL-33* expression or biological activity. Accordingly, the present findings demonstrate a genetic association rather than a causal or clinically predictive relationship. Sixth, the present candidate-gene study evaluated only five selected *IL-33* polymorphisms and did not comprehensively cover all genetic variation within the *IL-33* locus, including other variants previously associated with asthma susceptibility or *IL-33* expression. Larger multicenter studies with independent and ethnically diverse populations, together with functional investigations, are warranted to validate and extend these findings.

## 5. Conclusions

In conclusion, among the five *IL-33* polymorphisms investigated, rs1929992 was associated with asthma susceptibility in this Taiwanese population, with the C allele showing an association with lower asthma risk. The associations observed in the age-, sex-, and severity-stratified analyses should be regarded as exploratory and hypothesis-generating. Given the single-center design, moderate sample size, absence of independent replication, and lack of functional validation, the present findings do not establish rs1929992 as a clinically predictive biomarker. Larger independent and ethnically diverse studies, together with fine-mapping and functional investigations, are required to determine the reproducibility and biological basis of this association before any translational implications can be considered.

## Figures and Tables

**Table 1:** Distributions of baseline characteristics among the 198 asthmatic patients and 453 non-asthmatic controls.

Character		Controls (*n* = 453)		Cases (*n* = 198)		*p*-Value ^a^
		*n*	%	*n*	%	
Age (years)	25–40	285	63.4%	133	67.2%	
	>40	168	36.6%	65	32.8%	0.2972
Gender	Male	190	41.9%	83	41.9%	
	Female	263	58.1%	115	58.1%	0.9956
Smoking habits	Ever	127	28.0%	59	29.8%	
	Never	326	72.0%	139	70.2%	0.7161
Pulmonary functions	(mean ± SD)					
	FEV1/FVC (%)	80.8 ± 8.1		62.0 ± 13.0		<0.0001
	FEV1%	92.9 ± 5.8		69.1 ± 12.9		<0.0001
Symptoms severity						
	1 (mildest)			60	30.3%	
	2			65	32.8%	
	3			34	17.2%	
	4 (severest)			39	19.7%	

Abbreviation: FEV1, forced expiratory volume in first second; FVC, forced vital capacity; FEV1%, percent of predicted FEV1; ^a^ Chi-square with Yates’ correction test or Student’s *t*-test.

**Table 2:** Summary of the polymorphic sites, paired primer sequences, restriction enzymes, and expected DNA fragments after digestion.

Polymorphic Sites	Primer Sequences (5′→ 3′)	Restriction Enzymes	Genetic Variants	DNA Fragments, bp
rs1891385	Forward: CCACTCAAGACAGGTGTTGT Reverse: AATGGCTGGCAGGAACTGTT	*HpyCH4* V	A C	259 84 + 175
rs16924159	Forward: CTACAGACTTGATCCACTGG Reverse: GGTAGATTCCCTGGTTGAGC	*HpyCH4* V	A G	289 115 + 174
rs12551256	Forward: TCAGTCTTACAGAAGACGCC Reverse: CAGTGCTCAGTGAACAGAGA	*Dde* I	A G	383 160 + 223
rs1929992	Forward: TTGCAGCTGATGTACCCATC Reverse: ATTACAGGTGTGAGCCACCA	*Ssp* I	C T	395 197 + 198
rs7044343	Forward: AGCATGACAATGCCTGGTCA Reverse: CCAAGTTCAAGAGGCACTGA	*BsoB* I	T C	357 110 + 247

**Table 3:** Distribution of *interleukin-33* rs1891385, rs16924159, rs12551256, rs1929992, and rs7044343 genotypes among the controls and patients with asthma.

Genotype	Frequency, N (%)		OR (95%CI)	aOR (95%CI) ^a^	Nominal *p* ^b^	Bonferroni-Adjusted *p* ^c^
	Asthma (N = 198)	Non-Asthma (N = 453)				
rs1891385						
AA	101 (51.0)	247 (54.5)	1.00 (Reference)	1.00 (Reference)		
AC	84 (42.4)	182 (40.2)	1.13 (0.80–1.60)	1.16 (0.83–1.56)	0.5517	
CC	13 (6.6)	24 (5.3)	1.32 (0.65–2.70)	1.38 (0.71–2.61)	0.5586	
AC+CC	97 (49.0)	206 (45.5)	1.15 (0.82–1.61)	1.19 (0.84–1.55)	0.4582	
*p*_trend_					0.3516	
*p*_HWE_					0.1976	
rs16924159						
GG	125 (63.1)	276 (60.9)	1.00 (Reference)	1.00 (Reference)		
AG	67 (33.8)	160 (35.3)	0.92 (0.65–1.32)	0.94 (0.68–1.32)	0.7318	
AA	6 (3.1)	17 (3.8)	0.78 (0.30–2.02)	0.82 (0.38–1.91)	0.7785	
AG+AA	73 (36.9)	177 (39.1)	0.91 (0.64–1.29)	0.93 (0.66–1.26)	0.6568	
*p*_trend_					0.5399	
*p*_HWE_					0.2925	
rs12551256						
GG	53 (26.8)	133 (29.4)	1.00 (Reference)	1.00 (Reference)		
AG	99 (50.0)	223 (49.2)	1.11 (0.75–1.66)	1.14 (0.78–1.59)	0.6649	
AA	46 (23.2)	97 (21.4)	1.19 (0.74–1.91)	1.22 (0.75–1.86)	0.5493	
AG+AA	145 (73.2)	320 (70.6)	1.14 (0.78–1.65)	1.17 (0.79–1.63)	0.5624	
*p*_trend_					0.4644	
*p*_HWE_					0.8448	
rs1929992						
TT	75 (37.9)	118 (26.0)	1.00 (Reference)	1.00 (Reference)		
CT	90 (45.4)	234 (51.7)	0.61 (0.41–0.88)	0.65 (0.42–0.87)	0.0118	0.0590
CC	33 (16.7)	101 (22.3)	0.51 (0.32–0.84)	0.53 (0.34–0.81)	0.0101	0.0505
CT+CC	123 (62.1)	335 (74.0)	0.58 (0.40–0.82)	0.62 (0.43–0.79)	0.0032 *	0.0160 *
*p*_trend_					0.0036 *	0.0180 *
*p*_HWE_					0.4619	
rs7044343						
TT	61 (30.8)	130 (28.7)	1.00 (Reference)	1.00 (Reference)		
CT	104 (52.5)	240 (53.0)	0.92 (0.63–1.35)	0.95 (0.66–1.33)	0.7555	
CC	33 (16.7)	83 (18.3)	0.85 (0.51–1.40)	0.83 (0.54–1.39)	0.6063	
CT+CC	137 (69.2)	323 (61.3)	0.90 (0.63–1.30)	0.92 (0.66–1.28)	0.6523	
*p*_trend_					0.5138	
*p*_HWE_					0.1300	

N: number; OR: Odds ratio; CI: confidence interval. ^a^ aORs were estimated using multivariable logistic regression with adjustment for age, sex, and smoking status; ^b^ *p*-Value were calculated based on Chi-square test with Yates’ correction; ptrend: *p*-Value for Cochran-Armitage trend analysis; *p*_HWE_: *p*-Value for Hardy–Weinberg Equilibrium; Nominal *p*-Values < 0.05 are indicated for reference. After Bonferroni correction for five SNPs, * statistical significance was defined as a nominal *p*-Value < 0.01 or, equivalently, ^c^ Bonferroni-adjusted *p*-Value < 0.05.

**Table 4:** Allelic frequencies for *interleukin-33* rs1891385, rs16924159, rs12551256, rs1929992, and rs7044343 genotypes among the controls and patients with asthma.

Allelic Type	Frequency, N (%)		OR (95%CI)	aOR (95%CI) ^a^	Nominal *p* ^b^	Bonferroni-Adjusted *p* ^c^
	Asthma (N = 396)	Non-Asthma (N = 906)				
rs1891385						
A	286 (72.2)	676 (74.6)	1.00 (Reference)	1.00 (Reference)		
C	110 (27.8)	230 (25.4)	1.13 (0.87–1.47)	1.16 (0.88–1.43)	0.4036	
rs16924159						
G	317 (80.1)	712 (78.6)	1.00 (Reference)	1.00 (Reference)		
A	79 (19.9)	194 (21.4)	0.91 (0.68–1.23)	0.93 (0.71–1.22)	0.6012	
rs12551256						
G	205 (51.8)	489 (54.0)	1.00 (Reference)	1.00 (Reference)		
A	191 (48.2)	417 (46.0)	1.09 (0.86–1.38)	1.08 (0.83–1.36)	0.5006	
rs1929992						
T	240 (60.6)	470 (51.9)	1.00 (Reference)	1.00 (Reference)		
C	156 (39.4)	436 (48.1)	0.70 (0.55–0.89)	0.72 (0.57–0.90)	0.0044 *	0.0220 *
rs7044343						
T	226 (57.1)	500 (55.2)	1.00 (Reference)	1.00 (Reference)		
C	170 (42.9)	406 (44.8)	0.93 (0.73–1.18)	0.95 (0.74–1.21)	0.5695	

N: number; CI: Confidence interval; OR: odds ratio. ^a^ aORs were estimated using multivariable logistic regression with adjustment for age, sex, and smoking status; ^b^ *p*-Value were calculated; Nominal *p*-Values < 0.05 are indicated for reference. After Bonferroni correction for five SNPs, * statistical significance was defined as a nominal *p*-Value < 0.01 or, equivalently, ^c^ Bonferroni-adjusted *p*-Value < 0.05.

**Table 5:** Distribution of *interleukin-33* rs1929992 genotypes among asthmatic cases and non-asthmatic controls after stratification by age.

Genotype	25–40 Years, N		OR (95% CI)	aOR ^a^ (95% CI)	*p*-Value	>40 Years, N		OR (95% CI)	aOR ^a^ (95% CI)	*p*-Value
	Non-Asthma	Asthma				Non-Asthma	Asthma			
TT	72	55	1.00 (ref)	1.00 (ref)		46	20	1.00 (ref)	1.00 (ref)	
CT	149	59	0.51 (0.33–0.82)	0.53 (0.35–0.79)	0.0073 *^b^	85	31	0.84 (0.43–1.63)	0.82 (0.41–1.61)	0.7299 ^b^
CC	64	19	0.39 (0.21–0.72)	0.41 (0.22–0.73)	0.0040 *^b^	37	14	0.87 (0.39–1.95)	0.85 (0.36–1.91)	0.8953 ^b^
Total	285	133				168	65			
*p*_trend_					0.0010 *^c^					0.7071 ^c^

N: number; OR: odds ratio; CI: Confidence interval; ^a^ aORs were estimated using multivariable logistic regression with adjustment for sex and smoking status; ^b^ Based on Chi-square with Yates correction test; ^c^ Based on Cochran-Armitage trend analysis; * Statistically significant identified by *p*-Value less than 0.05.

**Table 6:** Distribution of *interleukin-33* rs1929992 genotypes among asthmatic cases and non-asthmatic controls after stratification by gender.

Genotype	Males, N		OR (95% CI)	aOR ^a^ (95% CI)	*p*-Value	Females, N		OR (95% CI)	aOR ^a^ (95% CI)	*p*-Value
	Non-Asthma	Asthma				Non-Asthma	Asthma			
TT	51	39	1.00 (ref)	1.00 (ref)		67	36	1.00 (ref)	1.00 (ref)	
CT	97	33	0.44 (0.25–0.79)	0.47 (0.27–0.82)	0.0082 *^b^	137	57	0.77 (0.47–1.29)	0.81 (0.51–1.33)	0.3933 ^b^
CC	42	11	0.34 (0.16–0.77)	0.35 (0.18–0.79)	0.0107 *^b^	59	22	0.69 (0.37–1.31)	0.71 (0.40–1.34)	0.3324 ^b^
Total	190	83				263	115			
*p*_trend_					0.0019 *^c^					0.2400 ^c^

N: number; OR: odds ratio; CI: Confidence interval; ^a^ aORs were estimated using multivariable logistic regression with adjustment for age and smoking status; ^b^ Based on Chi-square with Yates correction test; ^c^ Based on Cochran-Armitage trend analysis; * Statistically significant identified by *p*-Value less than 0.05.

**Table 7:** Genotype frequencies of *interleukin-33* rs1929992 genotypes in patients with milder and severer asthma.

Genotype	Milder Asthma (N) ^a^	Severer Asthma (N) ^b^	OR (95%CI)	aOR (95%CI) ^c^	*p*-Value ^d^
TT	42 (31.6%)	33 (50.8%)	1.00 (Reference)	1.00 (Reference)	
CT	63 (47.4%)	27 (41.5%)	0.55 (0.29–1.04)	0.57 (0.31–1.09)	0.0893
CC	28 (21.0%)	5 (7.7%)	0.23 (0.08–0.65)	0.27 (0.09–0.68)	0.0075 *
*p*_trend_					0.0024 *
TT	42 (31.6%)	33 (50.8%)	1.00 (Reference)	1.00 (Reference)	
CT+CC	91 (68.4%)	32 (49.2%)	0.45 (0.24–0.82)	0.46 (0.25–0.88)	0.0140 *
TT+CT	105 (79.0%)	60 (92.3%)	1.00 (Reference)	1.00 (Reference)	
CC	28 (21.0%)	5 (7.7%)	0.31 (0.11–0.85)	0.33 (0.13–0.87)	0.0303 *

N: number; CI: Confidence interval; OR: odds ratio; aOR: adjusted OR. ^a^ milder asthma: severe stages 1 and 2; ^b^ severer asthma: severe stages 3 and 4; ^c^ aORs were estimated using multivariable logistic regression with adjustment for age, sex, and smoking status; ^d^ Based on Chi-square test with Yates’ correction; *p*_trend,_ *p*-Value based on Cochran-Armitage trend analysis; * statistical significance is set as *p*-Value less than 0.05.

**Table 8:** Minor allelic frequencies of *interleukin-33* rs1929992 among different populations.

Polymorphism	Population	Sample Size of Controls, N	Minor Allele (C) Frequency
rs1929992			
	Global	148,910	0.3571
	European	78,494	0.3133
	African	41,440	0.3848
	American	15,258	0.4618
	East Asian	5158	0.4996
	Taiwanese (current study)	453	0.4812

Data were extracted from the NCBI dbSNP database [52], updated on 12 June 2026.

## Data Availability

The data that support the findings of this study are available on request from the corresponding author. The data are not publicly available due to privacy or ethical restrictions.

## Abbreviations

The following abbreviations are used in this manuscript:

IL-33	interleukin-33
OR	odds ratio
CI	confidence interval
PCR	polymerase chain reaction
RFLP	restriction fragment length polymorphism
HWE	Hardy–Weinberg equilibrium
FEV1	forced expiratory volume in one second
FVC	forced vital capacity
FEV1%	percent predicted forced expiratory volume in one second
GINA	Global Initiative for Asthma
PM2.5	particulate matter with an aerodynamic diameter ≤ 2.5 μm
IgE	immunoglobulin E
ILC2	group 2 innate lymphoid cell
SOCS3	suppressor of cytokine signaling 3
